# Nipah virus outbreak in Bangladesh: an enduring zoonotic threat

**DOI:** 10.1097/MS9.0000000000004319

**Published:** 2025-11-18

**Authors:** Muhammad Abdullah Ali, Fatima Sajjad, Zaryab Bacha, Hassaan Abid, Matti Ullah, Kamil Ahmad Kamil

**Affiliations:** a Department of Medicine, Khyber Medical College, Peshawar, Pakistan; b Department of Medicine, Indiana University School of Medicine, Indiana, USA; c Internal Medicine Department, Mirwais Regional Hospital, Kandahar, Afghanistan

Between January and August 2025, the International Health Regulations National Focal Point for Bangladesh notified WHO of four fatal Nipah virus (NiV) infections occurring in separate districts across three divisions, with no epidemiological links identified between cases. All infections were laboratory confirmed by RT-PCR and ELISA. Three of the cases had a history of consuming raw date palm sap, a well-established source of exposure, while the fourth – a child in Naogaon district – had no such history, with the source of infection remaining under investigation. This case was notable for occurring outside the usual December–April NiV season. In total, 305 contacts were identified across the 4 incidents, all of whom tested negative^[1]^.

Although only four laboratory-confirmed cases were detected, the occurrence of unrelated fatalities in different districts and even outside the typical Nipah season underscores the enduring threat posed by NiV and the need for continuous surveillance^[1]^. NiV is a zoonotic paramyxovirus maintained in fruit bats of the genus Pteropus. Transmission to humans occurs via contaminated food, animal contact, or person-to-person spread. In Bangladesh, the primary route of spillover is through consumption of fresh date palm sap contaminated by bat secretions^[2]^. Human infection ranges from asymptomatic disease to fatal encephalitis, often progressing rapidly to coma and death. No licensed therapeutics or vaccines exist, and case management relies on intensive support.

Case fatality ratios in outbreaks across South and South-East Asia range from 40% to 75%, and more recent episodes in Bangladesh have killed 71.7% of infected people. Recognizing its pandemic potential, the WHO classifies NiV as a priority pathogen for research and development of medical countermeasures^[3]^. Since its first detection in Bangladesh in 2001, NiV has caused nearly annual outbreaks. By September 2025, 347 cases had been recorded, with an overall case fatality rate of 71.7%^[4]^. Almost half of primary cases have been linked to date palm sap consumption, while approximately one-third resulted from direct person-to-person transmission. Although the yearly number of reported infections has remained below ten since 2016 (except in 2023), the virus’s persistently high lethality and repeated spillover underscore its epidemic potential.

WHO currently assesses the public health risk of NiV to be moderate at national and regional levels and low at the international level^[3]^. Nevertheless, recurrent spillovers across Bangladesh and multiple outbreaks in neighboring India – nine in Kerala state since 2018^[5]^ – show that the virus can cross borders and establish local transmission chains. In Bangladesh alone, 347 cases with a fatality rate of 71.7% have been recorded to date^[3]^. The ecology of NiV is closely tied to human activities and land-use changes that alter bat behavior and increase contact opportunities. Pteropus bats roost widely across Bangladesh and forage opportunistically in agricultural landscapes^[6]^. Cultural practices, such as harvesting and drinking raw sap, amplify exposure risk during the winter months when bat feeding coincides with the tapping season. Continued spillover in both typical and atypical seasons highlights the challenge of fully interrupting transmission. Bangladesh has developed robust Nipah surveillance since 2006, with sentinel sites, rapid response teams, and diagnostic capacity enabling early detection. Public health messaging discouraging raw sap consumption, along with community education, remains central to prevention. However, sustained engagement is needed, as the practice persists despite awareness campaigns. The 2025 cases demonstrate both the progress of national systems in rapidly identifying cases and the ongoing vulnerability of populations to recurrent spillover^[4]^. Repeated outbreaks in neighboring Indian states underscore that national borders do not constrain NiV. Heightened surveillance, research investment, and regional coordination under a One Health framework are essential to prevent future spillover events and accelerate development of vaccines and therapeutics^[5]^. This manuscript adheres to TITAN guidelines^[7]^.

## Data Availability

All data used are cited in the manuscript.

## Research registration unique identifying number (UIN)

Not required as it is Letter to the Editor.
